# Virus‐induced spore formation as a defense mechanism in marine diatoms

**DOI:** 10.1111/nph.16951

**Published:** 2020-10-25

**Authors:** Angela Pelusi, Pasquale De Luca, Francesco Manfellotto, Kimberlee Thamatrakoln, Kay D. Bidle, Marina Montresor

**Affiliations:** ^1^ Department of Integrative Marine Ecology Stazione Zoologica Anton Dohrn Villa Comunale Naples 80121 Italy; ^2^ Research Infrastructures for Marine Biological Resources Stazione Zoologica Anton Dohrn Villa Comunale Naples 80121 Italy; ^3^ Department of Marine and Coastal Sciences Rutgers University New Brunswick NJ 08901‐8520 USA

**Keywords:** *Chaetoceros socialis*, diatoms, life cycle, resting spores, single‐stranded RNA virus

## Abstract

- Algal viruses are important contributors to carbon cycling, recycling nutrients and organic material through host lysis. Although viral infection has been described as a primary mechanism of phytoplankton mortality, little is known about host defense responses.
- We show that viral infection of the bloom‐forming, planktonic diatom *Chaetoceros socialis* induces the mass formation of resting spores, a heavily silicified life cycle stage associated with carbon export due to rapid sinking.
- Although viral RNA was detected within spores, mature virions were not observed. ‘Infected’ spores were capable of germinating, but did not propagate or transmit infectious viruses.
- These results demonstrate that diatom spore formation is an effective defense strategy against viral‐mediated mortality. They provide a possible mechanistic link between viral infection, bloom termination, and mass carbon export events and highlight an unappreciated role of viruses in regulating diatom life cycle transitions and ecological success.

## Introduction

Many unicellular eukaryotes are characterized by heteromorphic life histories that include different morphological and physiological stages. Examples include organisms that have a haplo‐diplobiontic life cycle (von Dassow *et al*., 2009), can form multi‐cellular colonies or chains (Peperzak & Gäbler‐Schwarz, 2012), or have the capability to form quiescent or dormant stages such as resting cysts, spores, or akinetes (Ellegaard & Ribeiro, 2018). Heteromorphic life cycles can increase the range of conditions in which a species can survive or act as a defense strategy against predators and/or pathogens (Pančić & Kiørboe, 2018).

Resting stages are present in the life cycles of numerous species, and these species are widely distributed over the broad phylogenetic diversity of microalgae. Their distinctive features include the inability to reproduce (undergo binary fission), and a series of morphological and physiological traits such as thick cell walls, high concentrations of lipids and carbohydrates, and low metabolic activity, all of which allow for dormancy or quiescence for extended periods of time (Ellegaard & Ribeiro, 2018). The formation of resting stages has been linked to the onset of adverse environmental conditions such as lack of nutrients, changes in temperature or day length (Kuwata *et al*., 1993; Kremp *et al*., 2009), or food limitation for heterotrophic and mixotrophic species (Tillmann & Hoppenrath, 2013). In diatoms, resting spores play an important role in community structure and population dynamics, acting as ‘seed banks’ and allowing species to subsist under sub‐optimal conditions until more favourable environments return. The physiological transition to spores has also been implicated in mass export events following diatom blooms due to their heavily silicified, thick frustules that facilitate rapid sinking (Salter *et al*., 2012; Rynearson *et al*., 2013; Rembauville *et al*., 2016).

There are limited studies on the role of biotic interactions as triggers for resting stage formation. Infection of the dinoflagellate *Scrippsiella trochoidea* by the parasite *Amoebophrya* accelerated the formation of resting cysts that were resistant to further parasitic attacks (Chambouvet *et al*., 2011). Similarly, exposure of *Alexandrium ostenfeldii* to water‐borne chemical cues produced by the parasitic flagellate *Parvilucifera infectans* induced the formation of temporary cysts (Toth *et al*., 2004). Infection by viruses as a trigger for resting spore formation has not been extensively explored, in part because viruses have historically been considered to mediate the recycling of particulate organic matter through lysis of their host – a process referred to as the ‘viral shunt’ (Wilhelm & Suttle, 1999). However, recent findings suggest that viruses may also act as ‘shuttles’ of carbon to depth by altering host physiology, and enhancing the production of polysaccharidic, transparent exopolymer particles (TEP) (Nissimov *et al*., 2018) or proteinaceous coomassie staining particles (CSPs) (Yamada *et al*., 2018), which serve to facilitate the aggregation of ballasted phytoplankton (Laber *et al*., 2018). Given the aforementioned link between spores and export flux, a possible role of viral infection in spore formation could represent an unappreciated mechanism facilitating carbon flux out of the surface ocean.

Here, we investigated and quantified the dynamics of spore formation during viral infection of the marine planktonic diatom *Chaetoceros socialis* (Tomaru *et al*., 2009) using two host strains isolated from geographically distinct regions (Italy and Japan). We show that infection induces the transformation of vegetative cells into resting spores and that spores dramatically minimize the propagation of infectious viruses upon germination. Our results suggest that spore formation is a defense strategy against stressful biotic interactions with impacts for viral‐mediated carbon export and the ecological persistence of diatom populations in the face of pervasive viral pressure.

## Materials and Methods

### Maintenance of *C. socialis* hosts and virus

Two strains of *Chaetoceros socialis* Lauder were used in this study. *Chaetoceros socialis* strain APC12 was isolated in 2015 from a single short chain of cells that had germinated during incubations of surface sediments collected in the Gulf of Naples (Mediterranean Sea, Italy). The strain is preserved as dormant spores (Pelusi *et al*., 2019), and freshly established cultures were used for all experiments. Strain L‐4 (identified as *C. socialis* f. *radians*) was isolated in 2005 from Hiroshima Bay, Japan and kindly provided by Yuji Tomaru (National Research Institute of Fisheries and Environment of Inland Sea, Fisheries Research Agency). The D1–D3 region of the nuclear encoded large subunit ribosomal DNA of the two strains was sequenced as described in a study by Gaonkar *et al*. (2017) (L‐4 GenBank accession no. MT919894; APC12 GenBank accession no. MK765996). The two sequences were added to the alignment in the Gaonkar *et al*., (2017) study, and a maximum likelihood phylogenetic tree was constructed. Cultures were maintained in f/2 medium (Guillard, 1975), prepared with artificial seawater (ASW; Sigma‐Aldrich) containing a modified N : P molar ratio of *c.* 20 and a slightly higher silicate concentration (580 µM NO_3_, 29 µM PO_4_, 360 µM SiO_4_), at 18°C under a 12 h : 12 h, light : dark photoperiod at 80 µmol photons m^−2^ s^−1^. The virus used in this study was CsfrRNAV01, a single‐stranded RNA virus recently reclassified into the genus *Bacillarnavirus* (Vlok *et al*., 2019) and documented to specifically infect strain L‐4 (Tomaru *et al*., 2009). Virus lysates were generated by infecting cultures of strain L‐4 until host lysis, then removing cellular debris by filtration using a 0.22 µm pore‐size polycarbonate filter (Millex‐GV; Millipore). The resulting virus lysate had 1.70 × 10^6^ viral gene copies ml^−1^, as assessed with quantitative real‐time polymerase chain reaction (qRT‐PCR), and was stored at −80°C until use.

### Experimental set‐up

All experiments were carried out in triplicate at 18°C, with 180 μmol photons m^−2^ s^−1^, and a 12 h  :12 h, light : dark photoperiod. Cultures were grown to early exponential phase (*c*. 6 × 10^4^ cells ml^−1^), infected with the viral lysate (100 : 1, v/v) and monitored daily for cell concentration and Chl fluorescence. Uninfected cultures were maintained as controls. The concentration of both vegetative cells and spores was measured on fixed (1.6% final concentration of a neutral formaldehyde solution) subsamples of each replicate, using a Sedgewick‐Rafter chamber and a Zeiss AxioPhot microscope (Zeiss, Oberkochen, Germany) at ×400 magnification. Chlorophyll fluorescence (relative fluorescence units; RFUs) was measured at the same time of day using a Turner Designs fluorometer (model 10‐005R; Turner Designs Inc., Sunnyvale, CA, USA).

Nitrogen starvation, a known abiotic factor that induces spore formation (Pelusi *et al*., 2019), was used as control to test spore formation dynamics. Exponentially growing cultures were inoculated in triplicate flasks at an initial cell concentration of 3000 cells ml^−1^ in f/2 culture medium with low nitrogen content (23 μM nitrate). Cell and spore abundance was monitored for 5 d.

For germination experiments, cleaned spores (see the following section, ‘Isolating and cleaning spores’) were re‐suspended in f/2 medium in triplicate glass tubes and placed in the dark for 1 wk. They were moved to the light under standard experimental conditions and monitored for growth by Chl fluorescence.

### Isolating and cleaning spores

Cultures with microscopically‐verified spores were sonicated three times on ice for 1 min with a Branson 250 sonicator (100 W; Branson, Emerson Electric Co., USA) to break up vegetative cells into smaller pieces, and then filtered onto 1.2 μm pore‐size polycarbonate filters (RAWP04700; Millipore). Filters were washed three times with 50 ml of sterile ASW to remove any free viruses and cell debris. Samples were collected before and after washing and analysed via the most probable number (MPN) assay to determine the number of infectious viruses. A subsample was also collected to measure the concentration of clean spores.

### Quantifying virus abundance

Virus abundance was assessed using MPN to measure the number of infectious viruses (i.e. the number of viruses capable of infecting a new host) and qRT‐PCR to measure the number of viral gene copies. For MPN, individual wells in a 96‐well plate were filled with 240 μl of strain APC12 or L‐4 at cell concentrations of *c.* 3 × 10^5^ cells ml^−1^ using a Freedom EVO (Tecan, Männedorf, Switzerland) platform. A total of ten, 10‐fold serial dilutions were prepared from the viral lysate, and 10 μl of each dilution was transferred into eight wells containing the host culture. Inoculation with only growth medium served as a negative control; undiluted virus lysate served as a positive control. Microtiter plates were incubated at standard experimental conditions and checked daily with an Infinite M1000 Pro microplate reader (Tecan, Männedorf, Switzerland) until cultures infected with undiluted virus lysate became clear (no more than 15 d). Scores were defined as positive when samples were cleared; the number of infectious viral units ml^−1^ was calculated using the Environmental Protection Agency's Most Probable Number (EPA‐MPN) Calculator 2.0 (Klee, 1993).

For qRT‐PCR measurements of total extracellular virus concentration, 1.5 ml of culture was filtered through a 0.22 Sterivex GV filter unit (Millipore) and filtrates were stored at −80°C until nucleic acid extraction. For determination of intracellular viruses, cells or spores (*c.* 5.8 × 10^6^ total cells/spores) were collected onto 1.2 μm pore‐size polycarbonate filters (RAWP04700; Millipore) and then immediately submerged into 1 ml of TRIzol Reagent (Invitrogen) and stored at −80°C until nucleic acid extraction.

### Extraction of viral nucleic acids

For extracellular viral nucleic acid extraction, chloroform (1 : 50 v/v) was added to thawed lysates and mixed on a shaker for 10 min at room temperature (RT). The samples were centrifuged at 11 900 ***g*** for 20 min at RT, and the supernatant containing the viral particles was transferred into new microfuge tubes and treated with both RNase and DNase (1 μg ml^−1^ final concentration) for 30 min at 37°C to avoid contamination of nucleic acids from host cells. Viral particles were precipitated overnight at 4°C in an equal volume of 20% (w/v) polyethylene glycol (PEG) 8000 in 2.5 M NaCl. Samples were then centrifuged (11 900 ***g***; 30 min; 4°C) and the pellets were re‐suspended in 0.5 ml of TRIzol Reagent (Invitrogen) for RNA extraction, following the manufacturer's instructions. The resulting RNA pellet was dried, resuspended in RNase‐free water, treated with RNase‐free DNase I (Roche) and further purified on Qiagen columns (74104, RNeasy Mini Kit) following the manufacturers’ protocols. RNA was quantified with a NanoDrop ND‐1000 Spectrophotometer (Nanodrop Tecnologies Inc., Wilmington, DE, USA). A volume of 12 µl for each sample was used to synthesize cDNA trough qScript XLT cDNA SuperMix (Quanta BioScience) following the manufacturer’s protocol. Primer sequences specific to the gene encoding nonstructural polyprotein CSfrRV_gp1 were designed using sequence information available at the National Center for Biotechnology Information (NCBI; https://www.ncbi.nlm.nih.gov/gene/7559143) and primer3plus v.2.4.2 (Untergasser *et al*., 2012). The sequences for the forward and reverse primers are 5′‐TCGACAAGAACCAAGCACAG‐3′ and 5′‐GTCCCCAAAGTGGTTGAGAA‐3′, respectively, yielding a 192 bp PCR product. The procedure for extracting intracellular viral nucleic acids from host cells collected onto filters was the same as the procedure for extracellular viral nucleic acid extraction, with the only differences being that the amount of total RNA used for reverse transcription was 150 ng and the fact that the samples were not pre‐treated with RNase or DNase. To test whether the viral genome was inserted into the host genome as a stable DNA sequence (via lysogeny), we collected a subsample of viral‐induced spores, extracted DNA following the method outlined in a study by Gaonkar *et al*. (2017) and amplified it as described in the section that follows.

### Polymerase chain reaction and quantitative reverse‐transcription PCR (qRT‐PCR)

Polymerase chain reaction tests for the nonstructural polyprotein CSfrRV_gp1 contained the following reagents: 0.4 µM of forward and reverse primers, 2 µl of template, 5× XtraWhite buffer solution (GeneSpin, Milano, Italy), 0.2 mM of dNTPs, 1.12 U of XtraTaq Pol (GeneSpin) and water to a final volume of 20 µl. Thermal cycling conditions were as follows: 2 min at 95°C followed by 35 cycles of 15 s at 95°C and 30 s at 58°C, 30 s at 72°C and 7 min at 72°C. The PCR products were then run on an agarose gel (1.2%) and the resulting amplicons were purified using a GenElute gel extraction kit (Sigma) following the manufacturer's protocol. One amplicon fragment from both RNA and DNA source material was sequenced using an Ion Proton sequencer (Life Technologies) with an Ion P1 sequencing kit v.2 and searched against the GeneBank database.

The qRT‐PCR reactions contained the following: 5 µl of 2 × Power SYBR Green Master Mix (containing buffer, dNTPs, SYBR Green I, AmpliTaq Gold DNA Polymerase LD and Passive Reference; Thermo Fisher Scientific, Waltham, MA, USA), 0.7 µM of forward and reverse primers, 1 µl of template cDNA, and RNAse‐free water to a final volume of 10 µl. Reactions were performed on a ViiA 7 Real‐Time PCR System (Applied Biosystems, Life Technologies, Carlsbad, CA, USA) with 384‐Well Block using the following thermal cycling conditions: 10 min at 95°C, followed by 40 cycles of 15 s at 95°C and 1 min at 60°C, with the addition of a melting curve to assess the potential amplification of nonspecific products. Fluorescence was detected at the end of each cycle with the automatic determination of the threshold cycle (*C_t_*). A serial dilution of a viral reverse transcript sample was employed to construct a standard curve to quantify products. The viral copy number was calculated with empirical formulas which have been described previously (Tomaru & Kimura, 2016). Virus burst size was calculated by dividing the total number of viruses produced by the number of host vegetative cells lost (i.e. maximum host abundance minus final host abundance) as follows: (final virus concentration (*T_f_*) – initial virus concentration (*T*_0_))/(maximum concentration of vegetative cells in exponential phase before lysis – final concentration of vegetative cells after lysis phase).

### Transmission electron microscopy

Infected APC12 cells were collected for transmission electron microscopy (TEM) analysis using a previously described protocol (Zingone *et al*., 2006), with the exception that sections were stained with UAR‐EMS Uranyl Acetate Replacement Stain (Electron Microscopy Sciences, Hatfield, PA, USA) diluted 1 : 4 (v/v). Thin sections were examined with a Zeiss Leo 912 AB transmission electron microscope (Zeiss). Transmission electron microscopy preparations of extracellular virus‐like particles were made by adding chloroform (1 : 50 v/v) to lysates and incubating for 10 min at 37°C shaking at 600 rpm. The sample was then centrifuged (17 900 ***g***; 20 min; RT) to eliminate cellular debris. The resulting supernatant was mixed with an equal volume of 20% (w/v) PEG 8000 in 2.5 M NaCl and incubated overnight at 4°C. This sample was centrifuged (17 900 ***g***; 30 min; 4°C) and the pellet was resuspended in 10 μl of SM buffer (NaCl 100 mM; MgSO_4_·7H_2_O 8 mM; Tris‐Cl (1 M, pH 7.5) 50 mM, H_2_O). The virus suspension was mounted on a formvar‐carbon coated grid for 30 s, and excess solution was removed with blotting paper. The sample was stained for 10 s with 10 µl of 2% phosphotungstic acid. After the removal of excess dye, the grid was dried in a chemical hood for 2 d and examined on a Zeiss LEO 912 AB (Zeiss) microscope.

### Statistical analysis

A two–tailed Mann‐Whitney U test was used to evaluate the difference in concentrations of viral particles and infectious units at the end of the experiments, and the differences between the percentages of spores in infected and uninfected cultures of the two strains. Values were deemed significantly different when *P* < 0.05. Statistical tests were performed with GraphPad prism 6 (https://www.graphpad.com/scientific‐software/prism/).

## Results

### Host susceptibility to viral infection

**Fig. 1:**
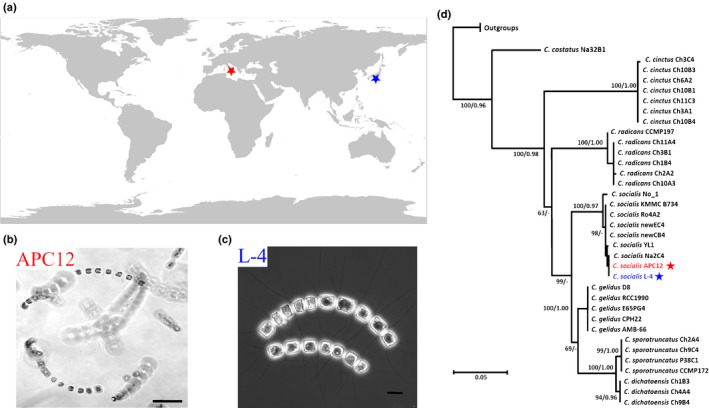
*Chaetoceros socialis* strains used in this study. (a) Isolation sites of strains of *C. socialis*, APC12 (red) and L‐4 (blue). (b) Globular arrangement of chains of vegetative cells of strain APC12. Bar, 50 µm. (c) Two chains of vegetative cells of strain L‐4. Bar, 10 µm. (d) Maximum likelihood tree inferred from partial 28S sequences of *Chaetoceros* species (Gaonkar *et al*., 2017), including *C. socialis* APC12 (red) and L‐4 (blue).

We tested the infection dynamics of two geographically distinct strains of *C. socialis,* one isolated from surface sediments in the Gulf of Naples, Mediterranean Sea, Italy (strain APC12) and the other from Hiroshima Bay, Japan (strain L‐4; Fig. 1a–c). Given the cryptic diversity reported for the *C. socialis* species complex (Chamnansinp *et al*., 2013; Gaonkar *et al*., 2017), we tested the genetic relatedness of these strains by sequencing the D1–D3 region of the nuclear encoded large subunit ribosomal DNA. The two sequences were 99% identical to the type sequence of *C. socialis* (strain YL1 in (Chamnansinp *et al*., 2013)) and were firmly placed within this species clade (Fig. 1d).

**Fig. 2:**
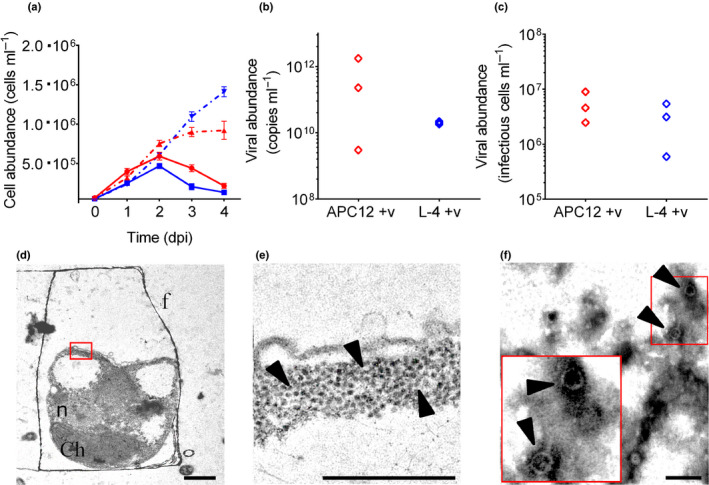
Virus infection dynamics of different *Chaetoceros socialis* strains. (a) Vegetative cell concentration (cells ml^−1^) in infected (solid line) and uninfected (dashed line) cultures of strain APC12, isolated from the Mediterranean Sea (red) and strain L‐4 isolated off coastal Japan (blue). Strains were infected with CsfrRNAV on day 0. Data shown are mean values ± SE from three independent experiments for APC12 and two experiments for L‐4, each with three replicates. (b) Viral gene copy number estimated by quantitative real time polymerase chain reaction (qRT‐PCR) at the end of a representative experiment (4 d post‐infection (dpi); *n* = 3) in APC12 (red) and L‐4 (blue). (c) Infectious units measured by most probable number (MPN) assay at the end of the same experiment (4 dpi) in APC12 (red) and L‐4 (blue). (d) TEM image of ultra‐thin section of an infected vegetative cell of strain APC12; chl, chloroplast; f, frustule; n, nucleus; bar, 1 µm. (e) Detail from (d) at higher magnification, demonstrating the presence of virus‐like particles (arrowed) in the vegetative cell; bar, 0.5 µm. (f) Free viruses in the lysate; bar, 0.1 µm.

The single‐stranded RNA virus CsfrRNAV was originally isolated with *C. socialis* strain L‐4 (Tomaru *et al*., 2009). Given the high species and, in some cases, strain specificity observed in other diatom host‐virus systems (Kimura & Tomaru, 2015), we tested whether CsfrRNAV could infect APC12 cells, with L‐4 serving as a positive control (Fig. 2a). Infection with L‐4 caused host demise at 3 d post‐infection (dpi) (Fig. 2a), consistent with previous observations (Tomaru *et al*., 2009). CsfrRNAV also successfully infected APC12 with similar dynamics to L‐4, causing a decline in host abundance 3 dpi. Both host strains produced a similar number of viruses, measured by either qRT‐PCR (*P* = 0.700) of the CSfrRV‐derived nonstructural polyprotein (CSfrRV_gp1) gene or most probable number (MPN) assay (*P* = 0.700), which respectively represent the total and infectious virus abundance (Fig. 2b,c). Upon host lysis, extracellular virus abundance increased four orders of magnitude for L‐4 and six for APC12, with an average of 1.98 × 10^10^ ± 1.05 × 10^9^ and 6.57 × 10^11^ ± 5.46 × 10^11^ viral gene copies ml^−1^, respectively (Fig. 2b). Infectious units for both strains were considerably lower (Fig. 2c), with average values of 3.05 × 10^6^ ± 1.40 × 10^6^ infectious units ml^−1^ in L‐4 (< 0.02% of the total viral abundance) and 5.34 × 10^6^ ± 1.92 × 10^6^ infectious units ml^−1^ in APC12 (< 0.03% of the total viral abundance). Burst size for strain L‐4 was 6.12 × 10^4^ and for APC12 was 1.18 × 10^6^. Transmission electron microscopy of ultra‐thin sections of infected strain APC12 confirmed the presence of virus‐like particles within vegetative cells (Fig. 2d,e), along with extracellular viruses, which were polyhedral in shape, with diameters of 20–22 nm (Fig. 2f). Collectively, these findings demonstrate that CsfrRNAV could successfully infect, replicate, and produce viral progeny in geographically distinct strains of *C. socialis*.

### Virus‐induced spore formation

**Fig. 3:**
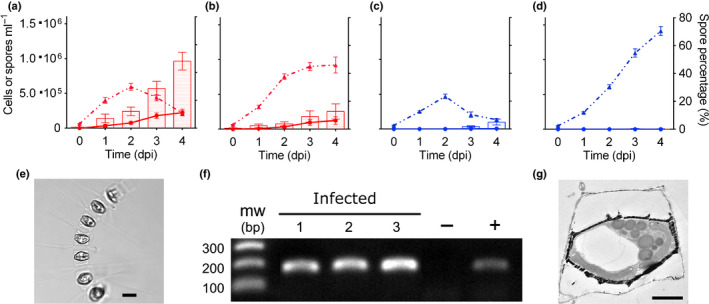
Viral infection induces spore formation in *Chaetoceros socialis*. (a, b) Abundance of vegetative cells (dashed line) and spores (solid line), and percentage of spores (bars) for strain APC12 (red) in infected (a) and uninfected (b) cultures. (c, d) Abundance of vegetative cells (dashed line) and spores (solid line), and percentage of spores (bars) for strain L‐4 (blue) in infected (c) and uninfected (d) cultures. Data shown are mean values ± SE for three independent experiments for strain APC12 and two experiments for strain L‐4, each of them with three replicates. dpi, days post‐infection. (e) Light micrograph of an AP12 chain of cells with visible resting spores inside the frustules of maternal cells; bar, 10 µm. (f) Polymerase chain reaction amplification of the nonstructural polyprotein CSfrRV_gp1 in RNA extracted from triplicate samples of virus‐induced spores (infected: 1, 2, 3) from a representative infection experiment. Minus symbol (−), uninfected vegetative cells in exponential growth phase; plus symbol (+) lysates used to infect cells. Sizes of molecular weight markers (mw) are indicated. (g) Ultra‐thin sections of a spore of the infected strain APC12; bar, 2 µm.

The drop in cell abundance during virus infection of APC12 was concurrent with a notable increase in spore formation compared to the uninfected control (*P* = 0.006), with an average of 48.1 ± 6.38% of cells transitioning to spores at 4 dpi (Fig. 3a,b). Induction of spores was much lower in infected L‐4 cultures, with only *c.* 5% of cells becoming spores at 4 dpi (Fig. 3c,d). To contextualize these findings, we tested the ability of both APC12 and L‐4 to form spores under nitrogen (N) limitation, a treatment known to induce a shift from vegetative cells to resting stages in most diatoms, including *C. socialis* (Pelusi *et al*., 2019). Less than 1% of the cells of strain L‐4 formed spores after 5 d in an N‐limited medium, compared to 99% ± 0.69 in strain APC12 (Supporting Information Fig. S1), indicating that L‐4 has a markedly lower ability to form resting stages than APC12. Given that APC12 represented a susceptible host strain capable of forming prominent spores, we further characterized the relationship between viral infection and spore formation in this strain.

**Fig. 4:**
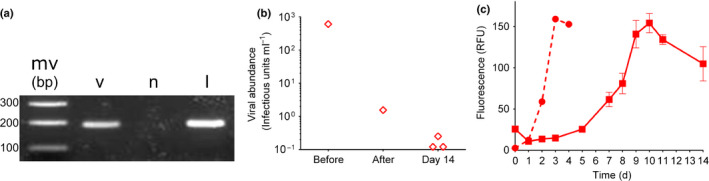
Spore formation represses viral propagation in *Chaetoceros socialis*. (a) PCR amplification of CSfrRV_gp1 from: cleaned, virus‐induced, spores (v); spores induced by nitrogen starvation (n); fresh virus lysate (l). Sizes of molecular weight markers (mw) are indicated. (b) Extracellular virus abundance before and after washing spores, and 14 d after germination (triplicate samples). (c) Growth curves of germinated cells obtained from infected spores (solid line) and N‐limitation (dashed line); data are mean values ± SE of three replicates.

We characterized the temporal dynamics of spore formation and host/viral abundance during infection (Fig. S2). Intracellular virus abundance increased at 2 dpi and reached a maximum of 1.22 × 10^4^ ± 9.08 × 10^3^ viral gene copies per cell on day 4. The initial increase in intracellular virus abundance coincided with a detectable increase in extracellular virus abundance, with the latter increasing to 1.10 × 10^8^ ± 4.02 × 10^7^ at 4 dpi (Fig. S2b). Spores were detected at 2 dpi, reaching nearly 80% of the culture by day 4. To determine whether viruses were associated with spores, we isolated cleaned spores on day 7 (see the Materials and Methods section) and performed qRT‐PCR. Amplification of CSfrRV_gp1 was detected in infection‐induced spores (Fig. 3e,f) with an average viral copy number of 9.17 × 10^4^ ± 2.27 × 10^4^ per spore. No amplification was observed in uninfected vegetative cells (Fig. 3f) or in spores generated by N‐limitation (Fig. 4a). We also assessed whether the ssRNA virus genome was integrated into the host genome, as has been observed for some RNA viruses (Ciuffi, 2016). We found no evidence of CsfrRV_gp1 amplification from host genomic DNA extracted from virus‐induced spores. Despite the high number of viral gene copies associated with spores, no mature virus‐like particles were observed via TEM (Fig. 3g).

The high intracellular viral abundance in both vegetative cells and spores led us to posit that infected spores may propagate viruses upon germination. A subsample of spores, which were confirmed to contain viral RNA (Fig. 4a), were collected and washed thoroughly to remove free, extracellular viruses before inoculation into fresh medium at a concentration of 1.1 × 10^4^ spores ml^−1^. MPN assays confirmed a 99.7% drop in extracellular viral abundance after washing (Fig. 4b), providing confidence that there was minimal carry‐over of free viruses. Spores germinated after 5 d, as indicated by an increase in Chl fluorescence, and grew until a peak abundance on day 10, after which Chl fluorescence decreased (Fig. 4c). Extracellular virus abundance measured on day 14 was barely detectable at only 0.16 ± 0.04 infectious units ml^−1^ (Fig. 4b), suggesting that the decrease in host abundance was not due to viral‐mediated mortality. Intriguingly, the dynamics of spore germination and growth were noticeably different than those for spores induced by N‐limitation, which germinated much faster, within one day, and peaked by day 3 (Fig. 4c). Taken together, these data suggest that virus‐induced spores are capable of germinating, albeit at a slower rate, but that they are not capable of transmitting infective viruses upon germination and likely harbour immature viruses that are incapable of replication and assembly.

## Discussion

We found that two geographically distant but genetically identical strains of *C. socialis,* APC12 (Mediterranean) and L‐4 (Hiroshima Bay, Japan), were both successfully infected by the ssRNA virus CsfrRNAV, which was originally isolated from strain L‐4. Infection dynamics were similar between the two strains, with host lysis observed at 3 dpi, and similar numbers of total (10^10^–10^11^ viruses ml^−1^, quantified by qRT‐PCR) and infectious (*c.* 10^6^ viruses ml^−1^, quantified by MPN) viruses produced. The much lower number of infectious viruses compared to the total (0.01–0.001%) was noteworthy and similar to observations made in other unicellular hosts (Van Etten *et al*., 1983; Cottrell & Suttle, 1995; Bratbak *et al*., 1998). This discrepancy remains an important consideration when contextualizing the potential ecological impact of total virus abundances in different systems. Despite similar viral production between the two host strains, the respective burst sizes (the number of total viruses produced per host cell) were markedly different; the burst size of APC12 was > 15‐fold higher than L‐4, suggesting viral replication was more productive in APC12. The mechanism behind enhanced intracellular replication in APC12 is unknown, but it resembles facilitated replication in plants and animals in which naïve hosts with no known previous exposure to a particular virus exhibit enhanced viral production upon infection (Jones, 2009).

Given that the majority of isolated diatom viruses have high species specificity, and in many cases, high strain specificity (Nagasaki *et al*., 2004; Tomaru *et al*., 2009; Arsenieff *et al*., 2019), these findings highlight that host–virus specificity is not regionally limited. Only one virus to date, CtenRNAV type‐II, has been shown to infect different *Chaetoceros* species (Kimura & Tomaru, 2015). However, strain specificity within a population is more commonly reported, as is the case with viruses that infect *C. debilis* (Tomaru *et al*., 2008) or *Guinardia delicatula* (Arsenieff *et al*., 2019). Infection of hosts isolated from different geographic areas has been observed in other microalgal species, such as the prasinophycean *Micromonas pusilla* (Zingone *et al*., 2006), the prymnesiophycean *Phaeocystis globose* (Baudoux & Brussaard, 2005), and the dinoflagellate *Heterosigma akashiwo* (Tai *et al*., 2003), but to our knowledge, this is the first report of a diatom virus capable of infecting conspecific strains from distant locations. Taken together, these findings highlight variable intra‐species specificity of algal viruses and illustrate the need for better understanding the mechanisms regulating host susceptibility and resistance (Short, 2012).

Abiotic environmental factors, such as light, temperature, and nutrient regime, are well‐known drivers of resting stage formation in unicellular eukaryotes (McQuoid & Hobson, 1996; Figueroa *et al*., 2018). Little is known about biotic interactions that trigger spore formation, in particular viruses, which are widespread and persistent biological stressors. Here, we observed that infection of the Mediterranean strain of *C. socialis* (APC12) induced up to *c.* 80% of the culture to form spores, demonstrating that viral infection can trigger spore formation in diatoms. The lack of appreciable spore formation in the Japanese strain (L‐4) in response to infection or N‐limitation suggests that either this strain has an intrinsically lower ability to form spores as compared to APC12 or that its ability has been reduced by prolonged time in culture. The impact of long‐term culturing on various life history traits has been widely observed across taxonomic groups, including dinoflagellates that have lost the ability to form resting cysts, diatoms that display reduced sexuality, and prymnesiophytes that have lost the ability to form a calcified diploid stage (Lakeman *et al*., 2009). Although a previous study qualitatively reported viral‐induced spore formation in L‐4, we note that spores were only observed at 23 dpi, well into nutrient limiting conditions and far outside of the time frame of lytic infection (Tomaru *et al*., 2009).

Regardless, our findings in APC12 demonstrate that viral infection can induce significant spore formation within days of infection, providing evidence for an unappreciated biotic trigger of resting stage formation in diatoms. Although the number of intracellular viral gene copies associated with spores was > 9‐fold higher than that of vegetative cells, the lack of visual evidence of mature virions within the cytoplasm suggests that upon infection, a significant pool of viruses started to replicate, but were unable to mature into viable virions upon transition of the host into spores. We posit that in the natural environment, heavily silicified spores induced by viral infection would rapidly sink out of the surface ocean, effectively removing a subpopulation from the water column and protecting it within sediments. ‘Infected’ spores could subsequently germinate under nutrient replete conditions, providing a seed population for future blooms.

The inability of spores to produce infectious viruses suggests the life cycle shift represents an effective defense strategy, allowing a large fraction of the population to avoid viral‐induced mortality. A viral‐induced life cycle transition has also been observed in the coccolithophore, *Emiliania huxleyi*, where the presence of large dsDNA *Coccolithoviruses* causes the host to shift from a susceptible, diploid, coccolith‐bearing phase to a resistant, haploid, noncalcified, motile phase (Frada *et al*., 2008, 2012). Virus infection also triggers the appearance of resistant, diploid or aneuploid morphotypes of *E. huxleyi* with lower growth rates than uninfected controls (Frada *et al*., 2017), an observation that is consistent with our findings of lower growth rates for *C. socialis* cells germinated from infected spores (Fig. 4c). The physiological mechanism(s) driving this indirect negative impact of viral infection on the growth and fitness of future diatom generations remains to be evaluated.

Abundant, widespread signatures of ssRNA viruses have been found throughout the world ocean (Miranda *et al*., 2016), and diatom viruses have been shown to actively infect natural diatom populations across different nutrient regimes (Moniruzzaman *et al*., 2017; Kranzler *et al*., 2019). The induction of spore formation by viral infection provides a novel mechanistic link between diatom bloom termination and mass export events attributed to heavily silicified spores with high sinking rates and aggregation (Rynearson *et al*., 2013), accompanying recent findings that virus infection may be a dominant driver of carbon export in the ocean (Laber *et al*., 2018; Kranzler *et al*., 2019). Although the subcellular mechanisms that regulate the induction of resting spores by viral attack remain to be clarified, our findings add to the ecophysiological and evolutionary arms race that governs host–virus interactions in the oceans (Frada *et al*., 2008, 2012; Bidle & Vardi, 2011; Bidle, 2015), with spore formation serving to preserve and propagate populations of hosts, while, at the same time, short‐circuiting the transmission of viruses to subsequent diatom generations.

## Author contributions

AP, KDB, KT and MM designed the study; AP, PDL and FM carried out the experiments; AP, KDB, KT and MM wrote the paper, which was discussed with all the authors.

## Supporting information


**Fig. S1** Spore formation in nitrogen limitation.
**Fig. S2** Time course of the abundance of cells, spores, and virus during infection of strain APC12.
Please note: Wiley Blackwell are not responsible for the content or functionality of any Supporting Information supplied by the authors. Any queries (other than missing material) should be directed to the *New Phytologist* Central Office.
