# Supplementary material for: Virus‐induced spore formation as a defense mechanism in marine diatoms
Source: New Phytol. 2020 Oct 25;229(4):2251–9. doi: 10.1111/nph.16951 (PMC7894508; doi:10.1111/nph.16951)
Supplement: Supplementary file 1 — Fig. S1 Spore formation in nitrogen limitation. Fig. S2 Time course of the abundance of cells, spores, and virus during infection of strain APC12. Please note: Wiley Blackwell are not responsible for the content or functionality of any Supporting Information supplied by the authors. Any queries (other than missing material) should be directed to the New Phytologist Central Office. [file NPH-229-2251-s001.pdf]

New Phytologist

***New Phytologist* Supporting Information**

Article title: **Virus-induced spore formation as a defense mechanism in marine diatoms**

Authors: Angela Pelusi[1], Pasquale De Luca[2], Francesco Manfellotto[1], Kimberlee Thamatrakoln[3*], Kay D. Bidle[3*], Marina Montresor[1*]

[1]Department of Integrative Marine Ecology, Stazione Zoologica Anton Dohrn, Villa Comunale, 80121 Naples, Italy
[2] Research Infrastructures for Marine Biological Resources, Stazione Zoologica Anton Dohrn, Villa Comunale, 80121 Naples, Italy
[3] Department of Marine and Coastal Sciences, Rutgers University, 08901 New Brunswick, New Jersey, USA

*Marina Montresor, e-mail: marina.montresor@szn.it Tel: x39 081 5833259
*Kimberlee Thamatrakoln, e-mail: thamat@marine.rutgers.edu Tel: x1 (848) 932-3464
*Kay D. Bidle, e-mail: bidle@marine.rutgers.edu Tel: x1 (848) 932-3467

Article acceptance date: 09 September 2020

The following Supporting Information is available for this article:

Fig. S1 Spore formation in nitrogen limitation.

Fig. S2 Time course of the abundance of cells, spores, and virus during infection of strain APC12.

Fig. S1 Abundance of vegetative cells (dashed line) and spores (solid line), and percentage of spores (bars) in response to N-limitation for strains APC12 (red) (a) and for strain L-4 (blue) (b). Data shown as mean ± standard error (n=3). The data for strain APC12 have been revised from figure 1e in Pelusi *et al*. (2019), reproduced here with permission.

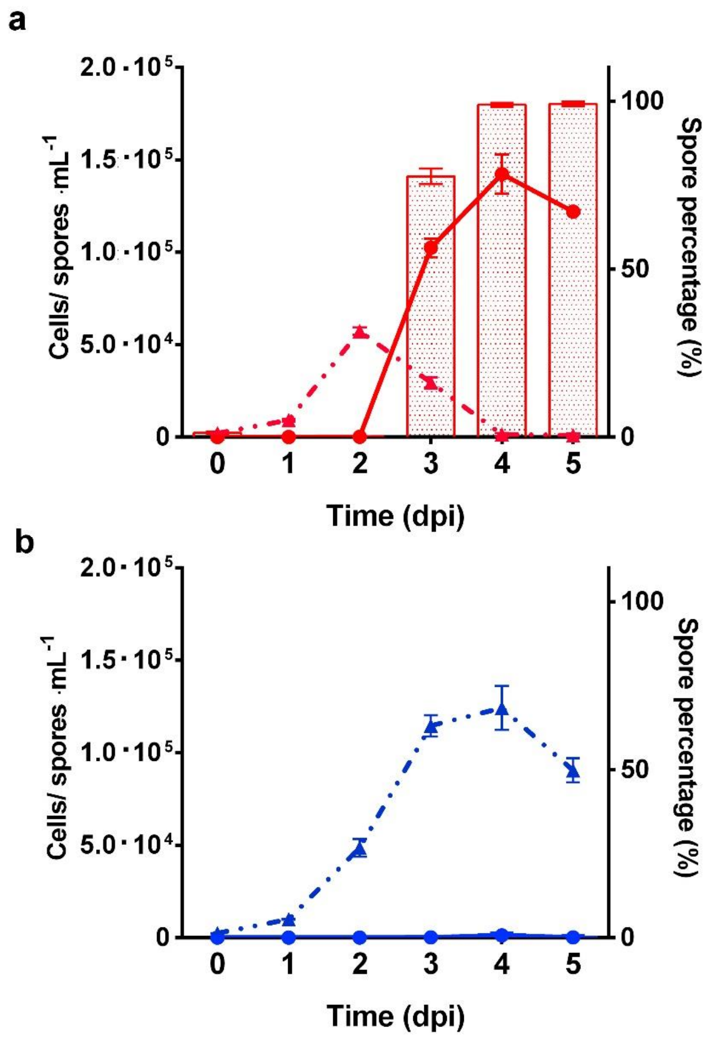

Fig. S2 (a) Abundance of vegetative cells (dashed line) and spores (solid line), and percentage of spores (bars) during virus infection. Data shown as mean ± standard error (n=3). (b) Extracellular (empty diamond) and intracellular (full diamond) viral gene copies per mL^{-1}. Data for day 0 represent the copy number of extracellular virus added at the start of the experiment. Note that the value reported for day 7 refers only to cleaned spores (see Materials and Methods and Results sections). Values shown as mean ± standard error (n=3).

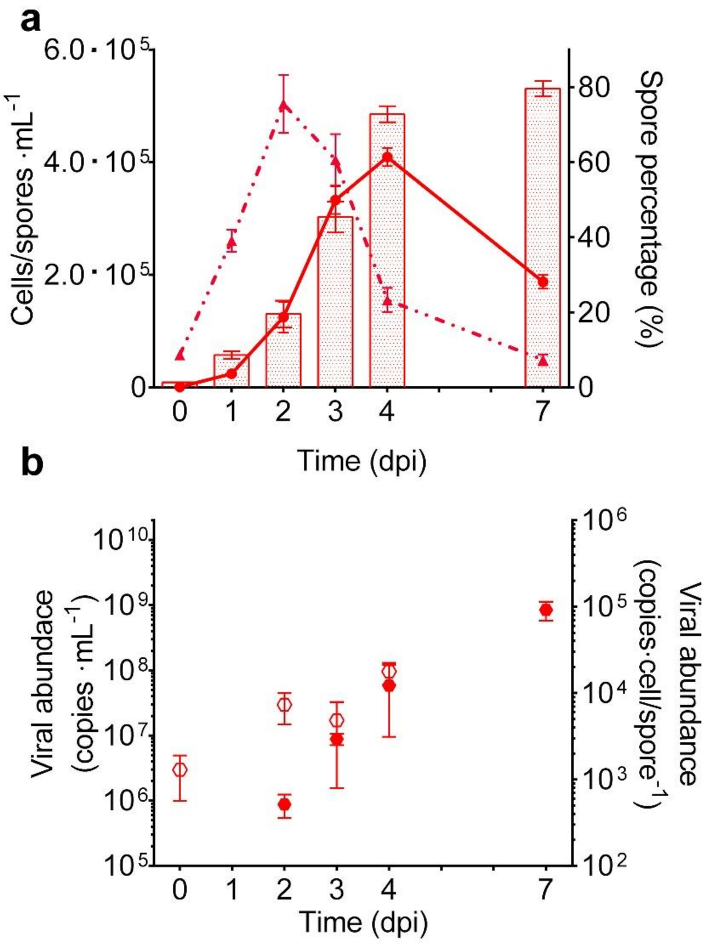

Reference

Pelusi A, Santelia ME, Benvenuto G, Godhe A, Montresor M. 2019. The diatom *Chaetoceros socialis*: spore formation and preservation. *European Journal of Phycology* **55**(1): 1-10.
